# Urodynamic profile in myelopathies: A follow-up study

**DOI:** 10.4103/0972-2327.48850

**Published:** 2009

**Authors:** Anupam Gupta, Arun B. Taly, Abhishek Srivastava, Murali Thyloth

**Affiliations:** Department of Psychiatric and Neurological Rehabilitation, National Institute of Mental Health and Neuro Sciences (NIMHANS), Bangalore, India; 1 Department of Neurology, National Institute of Mental Health and Neuro Sciences (NIMHANS), Bangalore, India

**Keywords:** Filling cystometry, myelopathy, neurogenic bladder

## Abstract

**Aims:**

To study the significance of filling cystometry in assessment and management of neurogenic bladder in myelopathies and correlate neurological recovery and bladder management in the follow up.

**Study Design:**

Retrospective analysis of reports of filling cystometry in patients with traumatic and non-traumatic myelopathy.

**Setting:**

Neuro-rehabilitation unit of a tertiary care university hospital.

**Methods:**

The study was carried out between September 2005 and June 2006 and included all subjects with myelopathy who underwent filling cystometry. ASIA impairment scale was used to assess neurological status during admission as well as in the follow up. Bladder management was advised based on the cystometric findings. Neurological recovery and mode of bladder management were correlated during the follow up after a minimum of 6 months.

**Results:**

Fifty-two subjects (38 males, 14 females), mean age 33.26 ± 14.66 years (10–80) underwent filling cystometry. Twenty patients had cervical, 24 had thoracic and 8 had lumbar myelopathy. Cystometric findings were overactive detrusor observed in 43 patients, (21 had detrusor sphincter dyssynergia (DSD), 22 without DSD) and areflexic/underactive detrusor in 9. Post-void residual (>15% of voided urine) was significant in 27 patients. Twenty-three patients (44%) reported for follow up (16 males, 7 females) after a mean duration of 9.04 ± 2.44 months (6–15 months). Neurological recovery was seen in 61% cases, while 1 patient showed deterioration. Only 26% patients reported change in bladder management during follow up. Correlation between neurological recovery and bladder management was found to be insignificant (*P* > 0.05) using spearman correlation co-efficient.

**Conclusions:**

Filling cystometry is valuable for assessment and management of neurogenic bladder after myelopathy. No significant relationship was observed between neurological recovery and neurogenic bladder management in the follow up in the present study.

## Introduction

Spinal cord lesions often cause neurogenic bladder dysfunction[1] and interfere with activities of daily living, travel, sleep and personal relationship of the patients. The recovery of detrusor function and its control are of major importance to patients with myelopathy, family members, and health care providers.[2] Spinal control of micturition is located at S2 to S4 level of the spinal cord. This correlates to vertebral levels of T12 to L2 but it may vary slightly in each individual.[3] Significant association exists between the level of a spinal cord lesion and its correlating bladder and sphincter behavior. Lesions above the spinal micturition center may lead to overactive detrusor and detrusor-sphincter dyssynergia (DSD), inducing reflex micturition with increased detrusor leak point pressures, causing incontinence and consequent renal damage if untreated.[4] Neurological tests, such as perianal pinprick sensation and the bulbocavernous reflex are moderately sensitive indicators of the return of bladder function after spinal cord injury. However, they are not predictive of the presence or absence of coexistent urodynamic abnormalities.[5]

It has been observed that when the patient is first seen in the peripheral health centers, usual practice is to manage bladder with indwelling catheter. It is imperative that urodynamic studies be performed at earliest and bladder is managed accordingly. It also helps in avoiding complications such as recurrent urinary tract infection and injury to the bladder, ureter and kidney.

This retrospective study was done to evaluate the significance of filling cystometry in assessment and management of neurogenic bladder in patients with myelopathy during initial admission and to observe in the follow up, whether neurological recovery is correlated with mode of bladder management/voluntary micturition.

## Materials and Methods

This study included 52 patients of myelopathy (traumatic and nontraumatic), who were receiving inpatient care in the Neurological Rehabilitation, Neurology or Neurosurgery unit. Patients younger than 7 years and with cognitive deficits were excluded from the study.

Filling cystometry was performed over a period of 9 months (September 2005 to June 2006) using multichannel pressure recording technology with Phoenix MK (Allbyn Medical, Scotland, UK) equipment. No patient had urinary tract infection or pyrexia at the time of study. Patients were advised bowel program on previous day and enema for bowel evacuation in the morning on the day of procedure. Study was performed using International Continence Society guidelines and urodynamicist was present at the time of procedure. Filling cystometry was performed with the patients lying in supine position on the urodynamic table. Prior to commencing the urodynamic study, bladder was evacuated voluntarily by the patient, and then post-void residual was measured by catheterization. Bladder filling was done with infusion of normal saline at medium fill rate (10–100 ml/ minute). Two-lumen catheter was inserted in the urethra (one for infusion of normal saline and other for recording intravesical pressure). A rectal catheter was inserted for recording of intra-abdominal pressure. Recordings of bladder sensations, pressures (intravesical, abdominal and detrusor) and compliance were made during the procedure. (Definitions according to International Continence Society).[6]

Patients were advised bladder management according to the observations made during the study with the goal to achieve a low-pressure reservoir and complete emptying. This management served as the baseline for subsequent urological evaluations. All patients were evaluated for neurological recovery as per American Spinal Injuries Association (ASIA) impairment scale before discharge. (ASIA impairment scale has 5 grades from A to E with A = Complete: No motor or sensory function is preserved in the sacral segments S4–S5, B = Incomplete: Sensory but not motor function is preserved below the neurological level and includes the sacral segments S4–S5, C = Incomplete: Motor function is preserved below the neurological level, and more than half of key muscles below the neurological level have a muscle grade less than 3, D = Incomplete: Motor function is preserved below the neurological level, and at least half of key muscles below the neurological level have a muscle grade of 3 or more and E = Normal: Motor and sensory function are normal).

Follow up assessment was done, minimum six months after the discharge, which included assessment of bladder management and re-evaluation of neurological recovery as per ASIA impairment scale. All patients were advised ultra-sound (US) abdominal scan, complete routine and microscopic urine examination, urine culture and sensitivity. Filling cystometry was not repeated in any case in the follow up.

Definitions of terminology used in urodynamics:

1. Filling cystometry is the method by which the pressure/volume relationship of the bladder is measured during bladder filling
2. Detrusor pressure is that component of intravesical pressure that is created by forces in the bladder wall (passive and active). It is estimated by subtracting abdominal pressure from intravesical pressure
3. Detrusor overactivity is a urodynamic observation characterized by involuntary detrusor contractions during the filling phase which may be spontaneous or provoked
4. Bladder compliance is calculated by dividing the volume change by the change in detrusor pressure during that change in bladder volume. It is expressed in ml/cm H_2_O
5. Cystometric capacity is the bladder volume at the end of the filling cystometrogram, when “permission to void” is usually given

## Results

Fifty-two patients of myelopathy (38 males, 14 females) with a mean age of 33.26 ± 14.66 years (10–80 years) were included in the study. Etiology of spinal cord lesions is mentioned in Table 1.

**Table 1:** Etiology of myelopathy

Diagnosis	No. of patients	Patients at follow up
Trauma	14	5
Transverse myelitis	10	6
Tumors	10	4
Peripheral intervertebral disk prolapse (PIVD)	7	2
Ossified posterior longitudinal ligament (OPLL)	4	2
OPLL with PIVD	2	2
Vasculitis	2	-
Pott's spine	2	1
Multiple sclerosis	1	1

Neurological symptoms varied from 9 to 1200 days with a mean of 199.44 ± 276.73 days and period of lower urinary tract (LUT) dysfunction varied from 8 to 950 days with a mean of 111 ± 190.19 days. Magnetic resonance imaging (MRI) scan of spine was performed in all the patients to determine the highest level and characteristic of lesion. Twenty patients (38.46%) had cervical, 24 (46.15%) had thoracic and 8 patients (15.38%) had lumbar spinal cord lesions. Twenty-seven patients (51.92%) had extra-dural lesions and 25 patients (48.08%) had intra-dural lesions. Twenty-two patients (42.3%) had focal and 30 patients (57.7%) had extensive lesions but no patients had multi-focal lesions. Neurological status was evaluated using ASIA impairment scale. It is mentioned in Table 2.

**Table 2:** Neurological status of the patients according to ASIA impairment scale

ASIA impairment scale	No. of patients		No change in follow up	Change in ASIA impairment scale at follow up (No. of Cases)						
	
	Initial	Follow up		A→C	A→D	B→D	C→D	C→E	D→E	D→C
A	6	0	0	1	1	3	7	1	1	1
B	4	0	0							
C	17	4	2							
D	24	17	6							
E	1	2	0							
Total	52	23	8				15		

Twenty-five patients (48.08%) were managing bladder with indwelling catheter at the time of urodynamics.

Filling Cystometry findings showed mean first sensation volume of 234.89 ± 120.67 ml and mean cystometric capacity of 401.44 ± 156.30 ml. Twenty-one patients had leak during the study and 27 patients had significant post-void residual urine (>15% of voided urine).

Eleven patients (21.15%) had high compliance, 20 (38.46%) had normal and 21 (40.38%) had low bladder compliance. Type of bladder according to urodynamics is mentioned in Table 3.

**Table 3:** Bladder classification according to detrusor activity after urodynamic study

Highest level of lesion (myelopathy)	No. of cases	Overactive detrusor		Areflexic/hypo active detrusor	
	
		Without DSD	With DSD	Without non-relaxing Sphincter	With non-relaxing Sphincter
Cervical	20	10	9	-	1
Thoracic	24	11	10	1	2
Lumbar	8	1	2	1	4
Total	52	22	21	2	7
		43		9	

Patients were advised bladder management according to urodynamic findings, which are summarized in Table 4.

**Table 4:** Advice on bladder management after urodynamics

Type of Neurogenic Bladder	Cervical	Thoracic	Lumbar	Bladder management advised
Overactive detrusor with DSD*	9	10	2	CIC**/ISC***, anti-cholinergic medication
Overactive detrusor without DSD	10	11	1	Timed voiding and anti-cholinergic medication
Hypoactive detrusor with non-relaxing sphinctor	1	2	4	CIC/ISC, Fluid restriction
Hypoactive detrusor with non-relaxing sphinctor	-	1	1	Voluntary micturition, timed voiding
Total	20	24	8	

* Detrusor-sphincter dyssynergia;

** Clean intermittent catheterization;

*** Intermittent self-catheterization

Twenty-three patients (44%) reported for follow up (16 males, 7 females). Mean follow-up duration was 9.04 ± 2.44 months (6–15 months). Out of these 23 patients, 4 (17.39%) had ASIA Grade C, 17 patients (73.91%) had ASIA Grade D and 2 patients (8.69%) had ASIA Grade E.

Mode of bladder management initially and during the follow up along with neurological status is summarized in Tables 5 and 6.

**Table 5:** Comparison of bladder management initially and at follow up

Method of bladder management	Cervical		Thoracic		Lumbar	
		
	Initial	Follow up	Initial	Follow up	Initial	Follow up
Voluntary micturition	-	2	-	3	-	-
Timed voiding (TV)	-	-	1	1	4	4
TV + anti-cholinergic (Ach)	3	3	1	-	1	1
CIC/ISC	-	1	-	-	-	-
CIC + Ach	5	2	7	5	1	1

Out of those 23 patients who reported for follow up, 14 (61%) showed neurological improvement [Table 6]. One patient (4%) of lumbar myelopathy had deterioration (from ASIA D to ASIA C during follow up).

**Table 6:** Neurological status and mode of bladder management at follow up

Level of lesion	No. of patients at follow up	Neurological improvement (ASIA Score)	Neurological deterioration (ASIA Score)	Mode of bladder management (M/m) at follow up		

				Change in M/m	No change in M/m
	23	14	1	-	-
Cervical		6	-	3	3
Thoracic		7	-	3	4
Lumbar		1	1	-	2

No significant correlation was found (*p* = 0.926) between neurological status and method of the bladder management (Using spearman correlation coefficient) as 50% of cervical myelopathy patients who showed improvement in neurological status were still following same bladder management method at follow up. Nearly 57% of thoracic myelopathy cases, which showed neurological improvement did not report any change in their mode of bladder management at follow up. One patient with lumbar myelopathy showed improvement and one patient with spinal tumor had deterioration in neurological status according to ASIA impairment scale at follow up but it is significant to note that neither patient reported change in their method of bladder management.

## Discussion

Historically, before urodynamic study with the coupling of cystometry and sphincter electromyography allowed the clinician to accurately diagnose DSD, mode of bladder management was chosen empirically, without reliance on objective testing.

The flaw with empirical therapy based on achieving a balanced bladder is that elevated intravesical pressure, responsible for the majority of urologic sequelae in patients with neurogenic lower urinary tract dysfunction, may be clinically silent. Indeed, low residual urine volume does not insure against severe urologic complications.[5]

The International Continence Society has published guidelines regarding filling cystometry, pressure-flow studies, and urethral pressure measurement in order to standardize the reporting of urodynamic results and technique.[7,8] The urodynamic manifestation of the neurogenic bladder dysfunction could be divided into three groups according to the category of the detrusor reflex activity: (a) overactive detrusor without dyssynergia, (b) overactive detrusor with dyssynergia and (c) detrusor areflexia.[9]

In our study, 82.7% patients showed overactive detrusor during filling cystometry with nearly half of them having had concurrent DSD. Remaining patients showed areflexic/hypoactive detrusor. The high number of overactive detrusor with or without DSD in our study was due to the fact that 85% of our patients had cervical or thoracic myelopathy, hence had supra sacral lesion in the spinal cord. Three cases of lumbar myelopathy showed overactive detrusor in urodynamics. One of these had myelopathy and two had cauda equina lesions. Patients with lesions of the cauda equina, far below the conus medullaris support the view that an overactive bladder may also be found in absence of an upper motor neuron lesion. It may be a consequence of decentralization of the parasympathetic ganglia situated within the bladder wall[10,11] or irritation of the lower sacral roots (as a “positive symptom” of the nerve lesion).

The treatment methods of the neurogenic bladder dysfunction include pharmacotherapy, training of the pelvic floor muscles, retention type catheter, sacral root electrical stimulation,[12,13] selective sacral root rhizotomy, pudendal nerve stimulation,[14] section of the external urethral sphincter[15] and insertion of the artificial urethral sphincter,[16] depending upon the type of bladder according to urodynamic evaluation. The goal is to maintain a low pressure, normal compliance of the bladder, eliminate the threat to the renal function and improve the quality of life.

Majority of patients in the study had incomplete cord lesion. This is understandable as nearly 70% patients had non-traumatic myelopathy in the study. Non-traumatic myelopathies more commonly present as incomplete cord lesions with thoracic and lumbar region as the more common site of lesion.

During follow up, 25% patients with cervical myelopathy were doing voluntary micturition with no backpressure changes. Three patients with cervical myelopathy, who were doing timed voiding (TV) along with anti-cholinergic medication, were doing same even in the follow up. Two out of 9 patients of thoracic myelopathy started doing voluntary micturition by the time of follow up and 5 patients were following same method. Out of 6 patients with lumbar myelopathy, no change was noted in the mode of bladder management at follow up. Four patients who were doing TV were still following same method in the follow up. No patient in the study, who was advised medication for bladder management at the time of discharge, stopped the drug. But voluntary micturition reported by some patients in place of CISC/TV (as advised by treating team at-discharge) in the follow up was started by them without consulting the treating team. However, all patients were advised abdominal ultrasound (KUB) to rule out significant post-void residual and back pressure changes in follow up. No patient with change in management method had significant PVR or BPC in abdominal ultrasound report. The patients' bladder management change was approved by the treating team only after obtaining normal reports.

Fourteen patients (61%) showed neurological improvement according to ASIA impairment scale during follow up. Six out of these 14 patients had cervical myelopathy and only 3 out of these 6 cases showed change in their method of bladder management. Similar trend was noted in patients with thoracic myelopathy as out of 7 cases that showed neurological improvement, only 3 showed change in the method of bladder management. One case of lumbar myelopathy had neurological recovery and other had deterioration. Interestingly, neither cases show any change in the method of bladder management.

### Limitations of the study:

This study had a few limitations. Patients had diverse etiology, level and completeness of myelopathies. Sample size is small and further, follow up was limited to 44% patients. Urodynamic study was not repeated in the follow up because of financial constraint and reluctance of the patients to stay in the hospital for the same.

## Conclusions

Spinal cord lesions are well known to cause neurogenic bladder dysfunction. Management of which, is of major importance not only for the patient but also for the caregivers. A significant association exists between the level of spinal cord lesion and the character of recovery of detrusor and sphincter function. Performing timely urodynamic procedure can best elucidate this association.

Filling cystometry is valuable in the diagnosis, classification, management and prognosis of the neurogenic bladder dysfunction. Proper bladder management according to the observations made during filling cystometry study helps in avoiding secondary complications in the urinary tract.

No significant relationship was observed between neurological recovery and mode of neurogenic bladder management in the follow up in this study.
